# Five years of malaria control in the continental region, Equatorial Guinea

**DOI:** 10.1186/1475-2875-12-154

**Published:** 2013-05-07

**Authors:** Andrea M Rehman, Andrea G Mann, Christopher Schwabe, Michael R Reddy, Irina Roncon Gomes, Michel A Slotman, Lee Yellott, Abrahan Matias, Adalgisa Caccone, Gloria Nseng Nchama, Immo Kleinschmidt

**Affiliations:** 1 MRC Tropical Epidemiology Group, London School of Hygiene and Tropical Medicine, London WC1E 7HT, UK; 2 Medical Care Development International (MCDI), New Hampshire Office, 104 Bradford Road, Keene, New Hampshire 03431, USA; 3 Department of Ecology and Evolutionary Biology, Yale University, New Haven, Connecticut, USA; 4 Department of Entomology, Texas A&M University, College Station, Texas, USA; 5 MCDI, Malabo Office, Bioko Island, Equatorial Guinea; 6 Ministry of Health and Social Welfare, Malabo, Equatorial Guinea

**Keywords:** Malaria, Equatorial Guinea, Long-lasting insecticide treated bed nets, Indoor residual spraying, Malaria indicator survey

## Abstract

**Background:**

A successful malaria control programme began in 2004 on Bioko Island, Equatorial Guinea. From 2007, the same multiple malaria interventions, though reduced in scope for funding reasons, were introduced to the four mainland provinces of Equatorial Guinea (the continental region) aiming to recreate Bioko’s success. Two provinces received long-lasting insecticidal nets (LLINs) and two provinces received biannual indoor residual spraying (IRS). Enhanced case management and communications were introduced throughout.

**Methods:**

Estimates of intervention coverage and indicators of malaria transmission for 2007 to 2011 were derived from annual malaria indicator surveys (MIS). Results were complemented by health information system (HIS) and entomological data. The personal protection offered by LLINs and IRS against *Plasmodium falciparum* infection was estimated with logistic regression.

**Results:**

The estimated proportion of children aged 1–4 using either an LLIN the previous night or living in a house sprayed in the last six months was 23% in 2007 and 42% in 2011. The estimated prevalence of *P. falciparum* in children aged 1–4 was 68% (N=1,770; 95% confidence interval [CI]: 58-76%) in 2007 and 52% (N=1,602; 95% CI: 44-61%) in 2011. Children 1–4 years had lower prevalence if they used an LLIN the previous night (N=1,124, 56%; adjusted odds ratio [aOR] 0.64, 95% CI: 0.55-0.74) or if they lived in a sprayed house (N=1,150, 57%; aOR 0.80, 95% CI: 0.62-1.03) compared to children with neither intervention (N=4,131, 66%, reference group). The minority of children who both used an LLIN and lived in a sprayed house had the lowest prevalence of infection (N=171, 45%; aOR 0.52, 95% CI: 0.35-0.78). High site-level intervention coverage did not always correlate with lower site-level *P. falciparum* prevalence. The malaria season peaked in either June or July, not necessarily coinciding with MIS data collection.

**Conclusions:**

Though moderate impact was achieved after five years of vector control, case management, and communications, prevalence remained high due to an inability to sufficiently scale-up coverage with either IRS or LLINs. Both LLINs and IRS provided individual protection, but greater protection was afforded to children who benefitted from both.

## Background

The population of Equatorial Guinea is exposed to one of the highest levels of malaria infection in the world [1,2]. A malaria control programme on the island of Bioko, covering two provinces of Equatorial Guinea, introduced multiple malaria interventions in 2004 and achieved large reductions in child mortality, infection and anaemia [3]. In the hope of repeating this success, from 2007 the same interventions, albeit at a reduced scale and scope, were introduced to the four mainland provinces of Centro Sur, Kie-Ntem, Litoral and Wele-Nzas, under the Equatorial Guinea Malaria Control Initiative (EGMCI). Vector control formed the basis of the initiative and consisted of indoor residual spraying (IRS) in Litoral and Kie-Ntem provinces and mass distribution of long-lasting insecticide treated nets (LLIN) in Centro Sur and Wele-Nzas provinces. Case management was improved in all four provinces through the introduction of ACT and other measures, and an integrated and supportive set of information, education and communications activities were promulgated. The initiative was funded largely by The Global Fund to Fight AIDS, Tuberculosis and Malaria (GFATM) with a start-up grant provided by the Marathon Oil Company Foundation to help jump-start activities prior to the finalization of the GFATM grant agreement and enhance the overall logistical capacity of the project. The government of Equatorial Guinea, in collaboration with the U.S. non-governmental organization Medical Care Development International (MCDI) implemented the initiative.

Data on intervention coverage and an evaluation of the impact the initiative had on malaria transmission in these provinces will be reported.

## Methods

### Study location

Equatorial Guinea is located on the West Coast of Central Africa at approximately 3° N latitude and 9° E longitude. It is bordered by Cameroon to the North and Gabon to the West and South. The country consists of seven provinces, four of which form the mainland or continental region, the location of the EGMCI. The continental region measures approximately 26,000 km^2^ in area. In 2011, its population was estimated to be almost one million (unpublished data, BIMCP; based on a 2.9% per annum increase from the 2001 population census estimate of three-quarters of a million). Equatorial Guinea has large off-shore oil reserves that began to be exploited extensively in 1997 resulting in a gross national income per capita of more than US$20,000 in 2011 [4] meaning the World Bank classify Equatorial Guinea as a high income country. Adult literacy is high (93% in 2011). In spite of extensive investments within the country in recent years, it remains under-developed with wealth unevenly distributed, and life expectancy at birth estimated to be 51 years. Equatorial Guinea was ranked 136 on the United Nations Human Development Index in 2011 [4]. The nation has had two rulers since gaining independence from Spain in 1968. The majority of people are of Fang ethnicity (of Bantu origin) and are Roman Catholic.

The wet seasons in the continental region runs from February to June and from September to December. Rainfall is highest on the coast, averaging 2,300 mm per annum in Bata whilst inland, in Mico Miseng, it is 1,500 mm. Temperature averages 26°C and the humidity is high year round. Malaria in the continental region is stable and hyper-endemic [5,6]. Transmission occurs year round at much higher levels than on the island of Bioko. The primary malaria vector is *Anopheles gambiae sensu lato* (*s.l.*), although three other species are also involved in transmission [7,8]. The use of LLINs and IRS was low before the EGMCI started [9].

### Interventions

**Figure 1:**
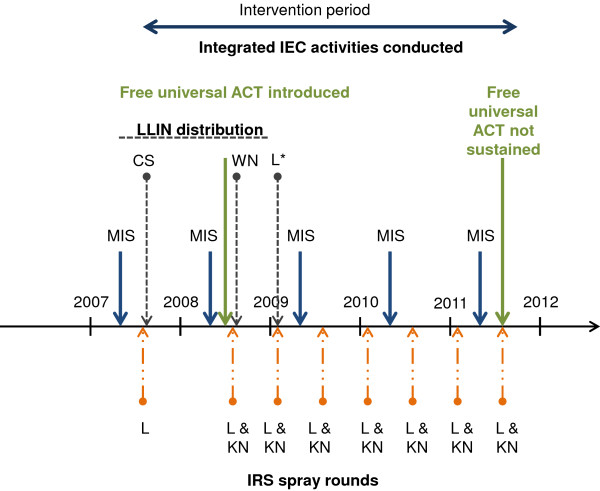
**Timing of malaria interventions, Equatorial Guinea 2007 to 2011.** A malaria indicator survey (MIS- blue solid lines) was carried out prior to implementation of any interventions in 2007 and then annually. Subsequently two provinces received interventions in 2007. Indoor residual spraying (IRS- lines with a long dash and two dots) commenced in Litoral (L, the coastal province) and long lasting insecticide treated bed nets (LLINs- dashed lines) were distributed in Centro Sur (CS, the province immediately east of Litoral). After the second MIS Artemisin combination therapy (Artesunate 50 mg + Amodiqaquine Hydrochloride 200 mg: denoted as ACT- green lines) was introduced region-wide and the remaining two provinces commenced vector control. Kie-Ntem (KN, the province in the far north east of the region) received IRS and Wele-Nzas (WN, the province in the south east of the region) received LLINs. Pregnant women attending ante-natal clinics region-wide received LLINs from 2009. Intervention activity stopped in August 2011. Information, education and communication campaigns ran throughout the intervention period as described in the text. Training for laboratory staff was carried out from 2009 until June 2011. *In water locked communities where IRS was not feasible.

The multiple malaria interventions that made up the EGMCI included both prevention through vector control and treatment through improved case management (Figure 1) as well as a comprehensive and integrated communications strategy designed to enhance knowledge, change behaviours and improve control-related practices.

PermaNet 2.0 (Vestergaard Frandsen) LLINs were distributed free-of-charge in Centro Sur Province from June through August 2007 and subsequently in Wele-Nzas Province from August through October 2008. A total of 146,608 nets were distributed. Quality control that was undertaken immediately after each of the LLIN mass distribution campaigns using Lot Quality Assurance Sampling (LQAS) [10] to identify lots where less than 90% of the houses had been visited by Red Cross volunteers, where less than 80% of the sleeping areas had received a net, and/or where less than 50% of the distributed nets were hung. Any lot failing either of these coverage targets were visited by a mop-up team. In addition to supply coverage, the LQAS surveys were used to evaluate usage rates among children less than five years of age. These surveys indicated that high supply coverage rates were rapidly achieved and usage rates increased to moderately high levels. For example, LQAS during LLIN distribution in Centro Sur in 2007 revealed that 66% children had slept under an LLIN the previous night. Similarly, in Wele Nsas the LQAS revealed that 60% of the children had slept under an LLIN the previous night. LLINs were later distributed in 40 villages in southern Litoral province (Kogo and Mbini sites) which had initially received IRS. These 40 villages were inaccessible for the annual MIS. Pregnant women attending antenatal clinics in all four provinces also received LLINs from 2009 through 2011.

A first round of indoor residual spraying was commenced in Litoral Province in June 2007 and was completed in December 2007. From March 2008 through September 2008, a second round of IRS was conducted in Litoral Province and a first round was conducted in Kie Ntem Province. From 2009 through 2011, due to a reorganization of the spray plan, two rounds of IRS were conducted in each province, the first occurring from February through March, and the second from August through September. A rotational insecticide scheme was used starting initially with four rounds of the pyrethoid insecticide Alpha Cypermethrin (Fendona™, Avima/BASF, South Africa and HI Kara, India), followed by one round of the carbamate insecticide bendiocarb (Ficam™, Bayer, South Africa), and ending with three rounds of the pyrethroid insecticide deltamethrin (K-Orthrin™, Bayer, South Africa) (Figure 1).

All four provinces were targeted for improvements to case management. Artemisinin-based combination therapy (ACT) was provided free-of-charge for uncomplicated malaria from June 2008 until August 2011 with Artesunate 50 mg + Amodiaquine Hydrochloride 200 mg (IDA foundation, The Netherlands; Arsuamoon™, Ghillin Pharmaceuticals Co.Ltd., China; Larimal™, IPCA Laboratories, India; and Coarsucum™, Sanofi Aventis, France). Patients suffering from severe malaria were also treated free-of-charge in accordance with the national treatment guidelines using injectable Artemether (Dafra Pharma GMBH, Switzerland). Diagnostic capacity was also improved. Rapid diagnostic tests (RDT; ICT™ Malaria P.f. Cassette ML01, R&R, Cape Town, South Africa) were distributed to health centres and health posts not equipped with a laboratory. In health centres with laboratories, and all hospitals, malaria diagnosis was confirmed by blood slide. Over the five-year duration of the project, 3,774 training sessions were conducted for individual service providers to improve and reinforce their diagnosis and treatment of uncomplicated and severe malaria.

Malaria prevention was promoted through comprehensive information, education and communication (IEC) messaging delivered through mass media, group-based activities at health facilities, and individualized communications through household visits conducted prior to every IRS round as well as prior to each of the two LLIN mass distribution campaigns. Malaria prevention in pregnancy was promoted among pregnant women attending ante-natal clinics held at Government health facilities, where they were offered two doses of intermittent preventive therapy (IPTp; Fansidar™, IDA foundation, The Netherlands) from three months gestation one month apart.

### Evaluation and statistical analysis

The initiative was evaluated by annual malaria indicator surveys (MIS) [11]. The results were complemented with data from the health information system (HIS) for 2009 and 2010 and entomological monitoring using light traps and human landing catches from 2007, 2009, 2010 and 2011 [12].

MIS were carried out at 17 sentinel sites between April and June of each year from 2007 to 2011. The sample size required for each MIS was determined based on an expected 30% reduction in malaria parasite prevalence among two to 14 year old children over the five-year initiative. Assuming a change in prevalence from 60% in year one to 42% in year five, with 80% power, 95% precision, a design effect of 1.5 between sentinel sites, and allowing for continuity correction, 191 children and 94 households were required per sentinel site. The sentinel sites were geographically spread throughout the four provinces so that there was at least one site per district. The exception was Bata district, the most populous, where there were five sites. Sentinel sites were: Akurenam, Bicurga and Niefang in Centro Sur province; Ebebeyin, Mico Miseng, and Nsok Nsomo in Kie-Ntem province; Ayamiken, Etofili, Kogo, Mbini, Ngolo, Ukomba and Yengue in Litoral province; Akonibe, Anisok, Mongomo, and Nsork in Wele-Nzas province (see map in [8]). Households were eligible for the survey if they included a pregnant woman or at least one child less than 15 years of age. Data were collected using Personal Digital Assistants (PDAs) in a survey programmed with Visual CE (SYWARE Inc., Cambridge, Massachusetts, USA) with data output to Microsoft Access® databases. Children under 15 years of age were tested for *P. falciparum* using RDT (ICT™ Malaria Combo Cassette ML02, R&R, Cape Town, South Africa) and had haemoglobin measured (HemoCue, Ängelholm, Sweeden), subject to parental consent. Anyone suspected of suffering from severe malaria was referred to and treated in hospital or clinic as appropriate according to national health guidelines.

The national HIS was co-ordinated by the Ministry of Health and Social Welfare (MOHSW) of Equatorial Guinea with technical and financial support from MCDI. During the EGMCI, data were captured from approximately 90% of the Government’s health facilities located in the four continental provinces; 36 health centres, nine district hospitals and four regional and provincial hospitals. Private health facilities do not report health information to the MOHSW. HIS data were entered on patient registers (outpatient, inpatient, ante natal care) printed and distributed by the EGMCI and folio copies of pages were collected on a monthly basis and entered centrally by MOHSW staffs in a Microsoft Access database. Approximately 10% of known folio copies were not received at the MOHSW. Suspected malaria cases were recorded in the HIS based on clinician diagnoses entered in patient registers. Confirmed cases were those with either a positive RDT or blood slide.

Entomological monitoring took place in all 17 sentinel sites. Mosquito specimens were initially collected on a monthly basis starting in December 2006 through August 2008 via a network of passive window traps installed in houses in each of the sentinel sites. Samples were sent to the Medical Research Council (MRC) South Africa for analysis. Collection volumes from these passive traps declined precipitously following the introduction of vector control activities, and ultimately were unable to produce sufficiently large quantities from which to reliably monitor infectivity levels. As such, starting in April 2009, trapping methods were changed and mosquito specimens were collected on a monthly basis, with the exception of September 2010 and Jan 2011, using light traps and human landing catches. Data collection ceased in June 2011. Specimens collected by light traps and human landing catches were sent to Yale University for analysis. Intact specimens captured using light traps and by human landing catch were analysed by PCR [13] and quantitative PCR (q-PCR) [14,15] to determine the presence of sporozoites and species identification [12]. In addition, gDNA aliquots extracted from window trap specimens (from January through September 2007) originally sent to MRC, were sent to Yale University for QPCR and PCR analysis. Bloodfed mosquitoes were excluded from testing for sporozoites to avoid specimens testing positive if they had ingested gametocytes. The sporozoite rate was calculated as the proportion of mosquitoes positive for sporozoites out of the total number tested.

Children under one were excluded from tabulations and analysis *a priori* as maternal antibodies offer some protection from malaria. Measures of intervention coverage and malaria transmission for children aged 1–4 are reported as many organizations including UNICEF focus on children under five [16]. Prevalence for children aged 2–14 is also reported because sample size calculations were based on this age range. Indicators which were derived from the MIS include, (1) LLIN use, (2) proportion of households where IRS was reported in the previous six months, (3) proportion of under 5 febrile cases receiving ACT, (4) proportion of women pregnant within the last year who received at least two doses of IPTp, (5) prevalence of infection with *P. falciparum*, (6) prevalence of moderate to severe anaemia (haemoglobin<8g/dL), (7) prevalence of reported fever (temperature≥37.5°C)) in the two weeks prior to the survey.

Analysis was carried out using Stata 12.1 software [17]. For the MIS, standard errors were adjusted to account for the survey design using the svy (survey) commands [18], where the primary sampling unit (PSU) was the sentinel site when reporting estimates combined over provinces and the household when reporting site level estimates. The effect of LLINs and IRS (living in a site targeted for these interventions or actually receiving them) on *P. falciparum* prevalence in children 1–4 years was determined by logistic regression, adjusting for survey year, household size and markers of socioeconomic status as individual covariates (access to a flush toilet, water source, light source, type of cooking fuel). Of these confounders, any for which the confidence interval included the null in the adjusted model, and which when removed did not change the main Odds Ratio (OR), were removed for the sake of parsimony. There were two kinds of missing data; data where status was reported unknown (in the case of IRS and LLIN use) and data where results were unavailable (in the case of a child not being present for an RDT or haemoglobin test).

### Ethics approval

This study was approved by the ethics committees of the Ministry of Health and Social Welfare of Equatorial Guinea and the London School of Hygiene and Tropical Medicine. Written informed consent was obtained from all parents or caregivers of the children who participated in the MIS. Anyone testing positive by RDT in the MIS was referred to a local clinic for appropriate treatment according to national policy.

## Results

### Malaria indicator surveys

In the five years of the initiative 8,741 households were surveyed (range 1,468-1,742 per year) and data from 8,741 children aged 1–4 were collected (range 1,523-1,894 per year). Half of the children were male (n=349 missing). It was not known whether 351 (4%) children aged 1–4 had slept under an LLIN the night prior to the survey. There were 654 (7%) children without an RDT result and 794 (9%) without a haemoglobin measurement. Most households contained 5–9 people (4,084; 50%), used a well as their water source (4,026; 49%), public electricity for their lighting (3,993; 49%), kerosene for their cooking fuel (3,350; 41%), and did not have a flush toilet (7,014; 86%). It was reported as unknown for 1,320 (16%) households if IRS was carried out in the last six months. Information was collected about 1,276 women currently pregnant and 1,803 women pregnant in the last year. RDT results were not recorded for 535 (42%) currently pregnant women.

By design, the targeted approach to delivering interventions meant that in general a given sentinel site received LLINs (Additional file 1) or IRS (Additional file 2) but not both. The reported use of an LLIN the previous night by children aged 1–4 was highest, 41% in 2009, in sites where LLINs had been very recently distributed but had waned by 2011 to only 13% in these sites (Additional file 1). Reported IRS coverage increased in sites in Litoral province in 2008, reflecting the first and only round of spraying carried out in Litoral in 2007, and in sites in Kie-Ntem province in 2009, reflecting the first and only round of spraying carried out in Kie-Ntem in 2008. Reported IRS coverage remained fairly stable from 2009 to 2011 when two rounds per year were conducted. Sites in Kie-Ntem province reported higher coverage than sites in Litoral province (Additional file 2) reflecting the constraints imposed by the GFATM preventing scale up in Litoral. The relationship between site level intervention coverage in a survey year and *P. falciparum* prevalence that year was variable. The highest site level percentage of children aged 1–4 reporting they slept under an LLIN the previous night was 59% in Akonibe in 2009 (Additional file 1). Estimated *P. falciparum* prevalence in children 1–4 in Akonibe that year was the lowest recorded in any site in 2009, 42% (Additional file 3). However, LLIN use at this site declined steadily in the two subsequent surveys (37% in 2010, 20% in 2011), which had no clear association with prevalence (70% in 2010 and 44% in 2011). The highest reported IRS coverage rates were achieved in Ukomba in Litoral Province and Nsok Nsomo in Kie-Ntem Province, 78% and 82% respectively, in 2009 (Additional file 2). For Nsok Nsomo, this coincided with a decrease in prevalence of *P. falciparum* in children 1–4 compared to previous years (Additional file 3). For Ukomba, however, the high IRS coverage in 2009 was observed at the same time as higher *P. falciparum* prevalence than the two previous years. The prevalence of *P. falciparum* was lowest in sites that were targeted for IRS compared to the sites targeted for LLINs (Additional file 3). The proportion of surveyed children aged 1–4 in all provinces combined using either an LLIN the previous night or living in a household sprayed in the last six months rose from a low of 23% in the baseline year to 54% in 2009, falling to 42% in the final survey year (Table 1) due to the substantial decline in LLIN utilization (Table 1).

**Table 1:** Indicators of intervention coverage obtained from MIS, [percentage, (95% CI), N]

**Indicator**	**Year**				
	**2007**	**2008**	**2009**	**2010**	**2011**
**Children aged 1–4 years reporting use of LLIN the previous night**	19.7%	19.9%	25.7%	18.1%	11.4%
	(16.1,23.9)	(16.5, 23.8)	(17.9, 35.5)	(13.7, 23.6)	(8.5, 15.1)
	1878	1507	1807	1536	1662
**Households reporting IRS in the last six months**	1.4%	12.3%	28.7%	26.5%	31.9%
	(0.7,2.9)	(5.7, 24.7)	(15.6, 46.8)	(15.3, 41.9)	(18.7, 49.0)
	1256	1398	1364	1380	1440
**Children aged 1–4 using an LLIN the previous night or living in a household reporting IRS in the last 6 months**	22.8%	32.3%	54.0%	46.1%	42.4%
	(18.8,27.5)	(25.4,39.9)	(46.3,61.6)	(36.0,56.5)	(29.3,56.7)
	1677	1349	1547	1333	1488
**Febrile cases aged 1–4 who received ACT**	26.7%	13.3%	16.5%	15.3%	45.2%
	(19.6,35.2)	(8.8,19.7)	(12.5,21.6)	(11.1,20.8)	(34.9,55.9)
	281	225	254	248	93
**At least two doses of IPT received during last pregnancy**	19.8%	13.1%	26.6%	11.3%	23.8%
	(14.2,26.9)	(9.9,17.1)	(23.2,30.2)	(8.4,15.0)	(18.2,30.4)
	425	375	335	327	341

Though EGMCI vector control interventions were targeted by province, a small proportion of children had access to both interventions at once. Children aged 1–4 years who lived in houses sprayed in the last six months and who slept under an LLIN the night before the survey had lower prevalence of *P. falciparum* (45%, n=171) than children with access to neither intervention (66%, n=4161, adjusted OR [aOR]: 0.52, 95% CI: 0.35-0.78). Prevalence was intermediate in children using LLINs alone (56%, n=1124) or living in an IRS treated house alone (57%, n=1150). Compared to those using neither intervention, children using IRS alone had 20% lower odds of parasitaemia (aOR 0.80, 95%CI: 0.62-1.03). Compared to those using neither intervention, children using LLINs alone had 36% lower odds of parasitaemia (aOR 0.64, 95% CI: 0.55-0.74). The dual protection of using both interventions was greater than that afforded by either IRS alone (aOR: 0.65, 95% CI: 0.43-0.98) or LLIN use alone (aOR: 0.82, 95% CI: 0.56-1.20).

**Figure 2:**
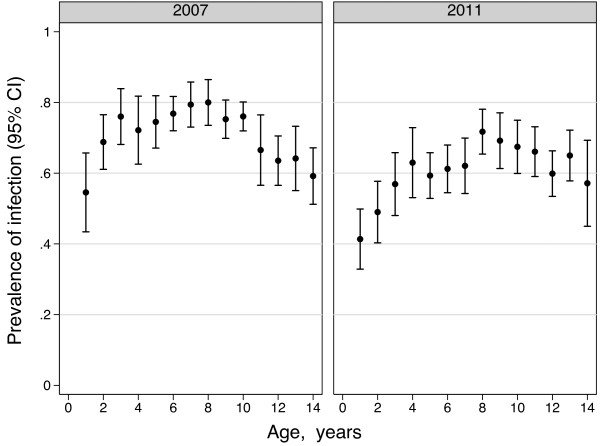
**Prevalence of P. Falciparum malaria and 95% confidence intervals by age in children under 15 years, Equatorial Guinea.** The prevalence of infection was highest among eight year olds in both 2007 and 2011.

Over the four provinces, indicators of malaria transmission were higher than had been anticipated for the sample size calculation at the outset of the study and fell, though remained high, by the end of the five-year initiative (Table 2). Prevalence was 72% (N=4143; 95% CI: 66-77%) among children aged 2–14 in 2007 and 61% (N=3318; 95% CI: 54-67%) in 2011. For children aged 1–4, the corresponding figures are 68% in 2007 and 52% in 2011 (Table 2). There was no shift in age of peak prevalence between 2007 and 2011; it remained at age eight (Figure 2).

**Table 2:** Indicators of malaria transmission obtained from MIS, [percentage, (95% CI), N]

**Indicator**	**Year**				
	**2007**	**2008**	**2009**	**2010**	**2011**
**Children aged 1–4 with *P. falciparum***	67.6%	63.1%	58.6%	70.0%	52.2%
	(58.0,75.9)	(54.2, 71.2)	(53.1, 63.9)	(63.2, 76.1)	(43.5, 60.8)
	1770	1334	1717	1664	1602
**Children aged 1–4 with anaemia (<8g/dL)**	10.1%	7.4%	7.6%	10.5%	7.3%
	(7.8,13.0)	(5.9,9.3)	(6.0,9.4)	(8.4,13.0)	(5.3,9.9)
	1731	1334	1717	1640	1525
**Children aged 1–4 reporting fever in the last two weeks**	15.0%	14.7%	13.9%	15.7%	5.6%
	(12.5,17.8)	(12.7,17.1)	(12.0,16.2)	(12.9,19.0)	(4.4,7.1)
	1878	1526	1821	1582	1664
**Pregnant women with *P. falciparum***	42.3%	40.6%	25.4%	45.8%	29.9%
	(34.2,50.8)	(30.3,51.8)	(17.3,35.7)	(37.5,54.4)	(22.4,38.7)
	258	64	122	120	177

One to four year olds reporting a fever in the last two weeks had significantly higher prevalence of *P. falciparum*, 73%, compared to those not reporting fever, 61% (crude OR:1.74: 95% CI: 1.47-2.06).

### Health facility and hospital data

**Figure 3:**
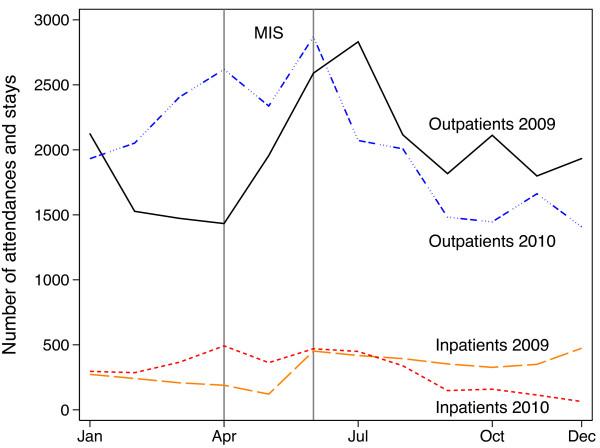
**Cases of outpatient attendances and inpatient admissions for malaria in children aged one to four, Equatorial Guinea.** Case numbers are those with diagnosed outcome of malaria recorded and may include repeat admissions for the one child. The grey vertical lines denote the period during which the annual malaria indicator survey (MIS) was carried out. In 2009 the MIS took place prior to the peak in case numbers, whereas in 2010 the MIS took place during the peak in case numbers. The figure shows the seasonality in case numbers.

The HIS database contained 347,722 outpatient attendances and 32,091 inpatient admissions for 2009 and 2010. The proportion of total attendances/admissions reported by each facility significantly differed between the two years. Children aged 1–4 accounted for 27% of outpatient attendances and 38% of inpatient stays. Half of these outpatient attendances were for malaria. Outpatient attendances and inpatient admissions were highest in July 2009 and in June 2010 respectively (Figure 3). The peak in attendances/admissions thus coincided with the MIS fieldwork in 2010 but not in 2009.

### Entomological data

**Figure 4:**
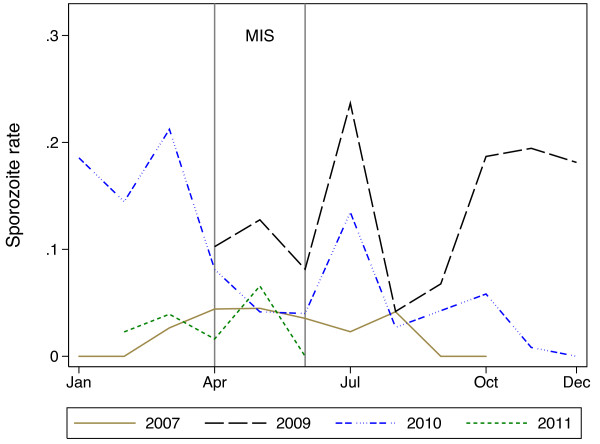
**Sporozoite rate by month and year for mosquitoes collected at 17 sentinel sites, Equatorial Guinea.** The grey vertical lines denote the period during which the annual malaria indicator survey (MIS) was carried out. The peak sporozoite rate in 2007 occurred during the MIS. Sporozoite rates in late 2009 and 2010 were very high. By 2011 the sporozoite rates had dropped down to 2007 levels again.

A total of 9,652 non-bloodfed mosquitoes were collected in the years 2007, 2009, 2010 and 2011. Data was available from eight sites in 2007, all 17 sites in 2009 and 2010 and 10 sites in 2011. Overall, 834 (8.6%) of the collected mosquitoes were sporozoite positive. To compare entomological data to the MIS and over the years of entomological monitoring a restricted dataset was used which covered the months of April to June in the years 2007, 2009, 2010 and 2011 in the eight sentinel sites which had data for all years. There were four sites from Litoral province (Kogo, Ngolo, Ukomba and Yengue), one from Kie-Ntem (Ebebiyen) and three from Wele-Nzas (Akonibe, Anisok and Mongomo). Comparing these months and years, there was a significantly higher sporozoite rate in 2009 (88/1144, 7.7%; 95% CI: 6.1-9.2%; p<0.01) compared to 2007 (58/1366, 4.3%; 95% CI: 3.2-5.3%). There was no evidence of a difference in sporozoite rate comparing 2010 (30/699, 4.3%; 95% CI: 2.8-5.8%; p=0.96) or 2011 (28/748, 3.7%; 95% CI: 2.4-5.1%; p=0.58) to 2007. The sporozoite rate significantly declined between 2009 and 2011 (p=0.001) using the restricted dataset. A peak in the sporozoite rate in July 2009 (Figure 4; which shows data from all sites) coincides with the peak seen in HIS outpatient attendances. The sporozoite rate in 2010 appeared to have two peaks, one before the MIS and one after the MIS, albeit continuing from the high level seen in 2009. The sporozoite rate dropped in April 2011 at the beginning of the final MIS survey; because collection of mosquito specimens ended in June 2011 it is not known if the peak sporozoite rate coincided with the MIS in 2011.

## Discussion

The five-year EGMCI sought to provide universal and free access to a comprehensive package of malaria control measures including either IRS or LLINs for vector control. Overall, due to limitations in funding and an inability to scale up control interventions to achieve at least 80% coverage with LLINs and/or IRS, the initiative appears to have achieved only a moderately positive impact on the estimated prevalence of *P. falciparum* in children aged 1–4, which fell by approximately 16% between 2007 and 2011. Vector control was shown to be effective at providing personal protection, with limited data suggesting greater protection was afforded to children who used LLINs and slept in IRS sprayed houses than those receiving only one of the two interventions. Vector control was also associated with reductions in the effective population size of mosquitoes [19] in four sites in the continental region. When looking across the five years of the initiative, the picture is more complex; the relationship between site-level intervention coverage and site-level *P. falciparum* prevalence in any given year was variable. Cross-sectional prevalence of *P. falciparum* in children aged 1–4 combined over the four provinces fell in 2008 and 2009 following the introduction of control interventions, rose in 2010 and then fell again in 2011 as the initiative came to an end. The continuous entomological monitoring suggested levels of *P. falciparum* rose late in 2009, which continued into 2010 and that there were two peaks in malaria in 2010. The second peak coincided with the peak seen in HIS cases in 2010.

The data presented suggests that the malaria season did not always peak at the same time each year and, therefore, might not have always coincided with the MIS. The apparent lower prevalence of *P. falciparum* in the 2011 MIS (compared to 2007) was not mirrored in the entomological data and may in fact be due, at least in part, to the MIS occurring prior to the peak malaria season. The age of peak prevalence not changing during the five years of the initiative may be further indication of lack of a major impact. The entomological data suggested that there was no change in sporozoite rates in vector mosquitoes between 2007 and 2011 which one might have expected to see if vector control had significantly reduced mosquito survival, albeit comparing specimens collected using different methods. There is strong evidence that vector control did have a significant impact on anopheles population size [19]. However, the data presented suggest that this did not have a measurable impact on malaria transmission using the presented indicators. It is possible that any impact on transmission could simply not be detected by these methods, over the limited time period for which the interventions were monitored.

The strength of this study is that data was used from five MIS which, aside from minor changes to questionnaires, were carried out systematically and consistently. Complementary additional data sources (HIS database and entomological data) supplemented and validated what can be gleaned from the MIS data. The main limitation of this study is the observational nature of the data available to evaluate the initiative. In addition, the sentinel sites of the MIS were not randomly chosen and refusal rates for the survey are not available, meaning that the sample may not be representative of the population. The targeted interventions were also not randomized to locations which could have introduced bias to any observed relationships. Apparent differences in LLIN use between LQAS samples and MIS estimates could be partly due to the lack of representativeness of MIS data or could be due to differences in the timing of surveys. The proportion of children with missing data for at least one of the analysed variables was 24% and was primarily due to the interviewee not knowing the IRS status of the household. Further, there were some differences in the patterns of missing data across years. It is difficult to assess the potential effect these missing data will have had on the presented results. This highlights the difficulties of working with self-reported measures of intervention coverage. For the HIS, there were differences in the proportion of attendances/admissions each health centre and hospital contributed to the total, by year. Approximately 10% of folio copies were not received for data entry so these observed differences could mean either that there were missing cases, or that there were true differences in health centre usage between years. Furthermore, because the HIS also did not contain data from private facilities the true number of malaria cases is likely to be much higher than reported here. However, any bias introduced by the missing HIS data from 2009 and 2010 is unlikely to have affected the apparent timing of the peak in malaria hospital visits across the two years.

The findings that both LLINs and IRS offered individual protection, after adjusting for indicators of socio-economic status, are consistent with the literature. LLINs have been shown to be effective for individuals [20] and when used in national control programmes [21]. The impact of IRS on malaria incidence has also been demonstrated although data from randomized controlled trials in areas of stable transmission are limited [22]. Evidence from other settings corroborates the finding that the combination of the LLINs and IRS offers greater protection than either intervention alone [23].

There were many barriers - related to funding the initiative as well as ecological and socio-economic – that prevented the initiative from achieving the big reductions in malaria seen on Bioko island (the Bioko Island Malaria Control Project [3]). Baseline prevalence of *P. falciparum* was much lower on Bioko (40% of 1–4 year olds in 2004, unpublished data, BIMCP) than in the continental region. The continental region experiences higher rainfall than Bioko and has a large number of malaria vector species [8]; the geographical area of the continental region is also much greater than that of the island. People are poorer than on the island, they less commonly have access to a flush toilet, and the houses are of poorer construction, with a greater proportion of houses having open eaves (unpublished data, EGMCI and BIMCP). Before the start of the EGMCI, *Anopheles gambiae* were shown to have a much higher level of the *kdr* mutation than those on Bioko, suggesting that the elevated levels of *kdr* alleles among mosquitoes collected in the continental region could have rendered them less susceptible to the pyrethroid insecticides used in LLINs and extensively by the EGMCI for IRS [8]. Analysis of mosquitoes caught in the period 2009–2011 showed that the level of *kdr* mutation increased over the period of the initiative [12] although the authors commented that high *kdr* in itself would not necessarily make LLINs or IRS cease to be effective. Other authors have shown susceptibility of mosquitoes to pyrethroid insecticides in Equatorial Guinea [24]. This could suggest that the poor impact of the current study was unlikely to be the result of insecticide resistance. Operationally, the Bioko programme covered the entire island with two rounds of IRS per year starting in the first year of the project, introduced island-wide free ACT for treatment starting in the second year, and distributed and helped hang LLINs island-wide in the third year. In comparison, the funding provided for the EGMCI limited vector control to either IRS or LLIN but not both in the same province. In those provinces that received IRS, coverage was constrained by lack of resources to target 80% of the houses (the original plans and budget were formulated on an estimate of the number of houses which later proved to be substantially underestimated) as well as by an inability in 2007 and 2008 to spray more than one time during the year (leaving houses effectively unprotected due to the limited residual life of the pyrethroids insecticide applied). In provinces that received LLINs, intra-familial demand for nets in non-recipient provinces is believed to have led to a substantial intra-family and inter-provincial reallocation of LLINs. This coupled with usual wear and tear and diminished residual insecticidal efficacy rapidly reduced the effective coverage and protection offered by the LLINs distributed by the EGMCI. Moreover, the lack of financing to replenish nets (other than through routine ante natal care outlets) meant that it was not possible to sustain coverage even at the moderately high coverage levels achieved immediately after the mass distribution campaigns.

While it is not possible to make definitive pronouncements, the data presented here suggest that the inability to achieve high effective coverage with at least one vector control intervention, much less offering simultaneous high coverage with both LLINs and IRS, prevented achieving a major impact on parasite prevalence in a setting of intense malaria transmission, such as mainland EG. In a high transmission setting such as Equatorial Guinea, the World Health Organization recommends a universally targeted package of interventions [25] with coverage scaled-up to levels that exceed those attained under the EGMCI. While LLINs may be seen as the preferred intervention to achieve universal coverage given the operational challenges imposed by IRS [25], the rapid loss rate evidenced when LLINs are not universally distributed as occurred on mainland Equatorial Guinea, or associated with wear and tear or repetitive washings which reduce the protective efficacy of the nets, suggest that LLINs alone are unlikely to be sufficient either to achieve the desired impact. Expanding the scope of vector control to include both LLINs and IRS and ensuring that together they are scaled up sufficiently to at a minimum ensure that at least 80% of households are protected by one or the other, may be the preferred approach. Evidence from Bioko suggests that in settings of year-round transmission there are benefits to providing high simultaneous coverage with LLINs and IRS if the residual life of insecticides used for IRS cannot assure year-round protection [26]. Similarly, in contexts where LLIN loss rates are high, there are likely to be benefits from simultaneous application of IRS as well.

## Conclusions

Due to funding limitations which precluded achieving the requisite scale and scope of control interventions, as well ecological and socio-economic factors associated with the high year-round transmission levels, the EGMCI achieved only moderate impact in reducing the prevalence of infection in the continental region of Equatorial Guinea. The initiative sought to provide universal and free access to IRS or LLINs in all study sites in addition to improving case management and informing and educating the population on how to best prevent transmission. Vector control was shown to be effective at providing individual-level protection from LLINs or IRS alone, though in this setting of intense transmission greater protection was afforded to children who benefitted from both LLINs and IRS. In spite of an observed overall decrease in *P. falciparum* prevalence between the pre-intervention period and the final year of the project, prevalence remains high, and the relationship between intervention coverage and *P. falciparum* prevalence in different sites throughout the continental region was inconsistent across the study years. Equatorial Guinea was recently the first sub-Saharan country to be declared a high income country making it no longer eligible for funding from the Global Fund. The challenge is, therefore, for the EG national malaria control programme to re-instate and intensify the interventions that were carried out under the EGMCI and which have now ended. The lesson of the EGMCI is that unless these interventions are rolled out on a high enough scale and with sufficient scope over a sustained period, any impact will be limited in magnitude and any gains can be quickly reversed once the interventions cease. The EGMCI experience indicates that to achieve the Government’s goal of replicating the successes of the Bioko Island Malaria Control Project throughout Equatorial Guinea, it will initially need to commit sufficient oil revenues to scale up for impact by extending the coverage and expanding the scope of the EGMCI until it achieves pre-elimination prevalence levels.

## Abbreviations

ACT: Artemisinin-based combination therapy; BIMCP: Bioko Island Malaria control programme; EGMCI: Equatorial Guinea Malaria Control Initiative; HIS: Health information system; IPT: Intermittent preventive therapy; IRS: Indoor Residual Spraying; LLIN: Long lasting insecticide-treated bed net; MIS: Malaria Indicator Survey; RDT: Rapid diagnostic test.

## Competing interests

CS, IRG, AM and LY are employed by the international NGO Medical Care Development International (MCDI) which was supported by a consortium led by the Global Fund with supplementary financial support from the Marathon Foundation to carry out malaria control on the mainland of Equatorial Guinea. GNN is employed by the government of Equatorial Guinea.

## Authors’ contributions

AMR and AGM conceived the idea, drafted the paper and carried out the statistical analysis. IK and CS designed the study and provided extensive critical input into the draft and interpretation of the data. MRR, MAS and AC provided entomological data and interpreted the entomological data in context. LY, IRG, AM and GNN ran the study and provided critical input into the draft and interpretation of the data. All authors read and approved the final version of the manuscript.

## Supplementary Material

Additional file 1 **Use of LLIN the previous night in children aged 1-4 (percentage, [95% CI], N), Equatorial Guinea.** Description: Data on the use of LLIN the previous night among children 1-4 years old broken down by sentinel site, province and by targeted intervention. Click here for file

Additional file 2 **Percentage of households reporting IRS in the last six months (percentage, [95% CI], N), Equatorial Guinea.** Description: Data on household IRS coverage broken down by sentinel site, province and by targeted intervention. Click here for file

Additional file 3 **Prevalence of P. falciparum in children aged 1-4 (percentage, [95% CI], N), Equatorial Guinea.** Description: Data on prevalence of P. falciparum in children aged 1-4 years broken down by sentinel site, province and by targeted intervention. Click here for file

## References

[B1] Gething PW Patil AP Smith DL Guerra CA Elyazar IRF Johnston GL Tatem AJ Hay SI A new world malaria map: *Plasmodium falciparum* endemicity in 2010 Malar J 2011 10 378 10.1186/1475-2875-10-378 22185615 PMC3274487

[B2] Cibulskis RE Aregawi M Williams R Otten M Dye C Worldwide incidence of malaria in 2009: estimates, time trends, and a critique of methods PLoS Med 2011 8 e1001142 10.1371/journal.pmed.1001142 22205883 PMC3243721

[B3] Kleinschmidt I Schwabe C Benavente L Torrez M Ridl FC Segura JL Ehmer P Nchama GN Marked increase in child survival after four years of intensive malaria control AmJTrop Med Hyg 2009 80 882 888 PMC3748782 19478243

[B4] International Human Development Indicators, Equatorial Guineahttp://data.worldbank.org/country/equatorial-guinea

[B5] Rehman AM Coleman M Schwabe C Baltazar G Matias A Gomes IR Yellott L Aragon C Nchama GN Mzilahowa T Rowland M Kleinschmidt I How much does malaria vector control quality matter: the epidemiological impact of holed nets and inadequate indoor residual spraying PLoS One 2011 6 e19205 10.1371/journal.pone.0019205 21559436 PMC3084796

[B6] Cano J Descalzo MA Moreno M Chen Z Nzambo S Bobuakasi L Buatiche JN Ondo M Micha F Benito A Spatial variability in the density, distribution and vectorial capacity of anopheline species in a high transmission village (Equatorial Guinea) Malar J 2006 5 21 10.1186/1475-2875-5-21 16556321 PMC1435759

[B7] Molina R Benito A Roche J Blanca F Amela C Sanchez A Alvar J Base-line entomological data for a pilot malaria control program in Equatorial-Guinea J Med Entomol 1993 30 622 624 8510123 10.1093/jmedent/30.3.622

[B8] Ridl FC Bass C Torrez M Govender D Ramdeen V Yellot L Edu AE Schwabe C Mohloai P Maharaj R Kleinschmidt I A pre-intervention study of malaria vector abundance in Rio Muni. Equatorial Guinea: their role in malaria transmission and the incidence of insecticide resistance alleles. Malar J 2008 7 194 10.1186/1475-2875-7-194 18823554 PMC2564967

[B9] World Health Organization Africa Region Stratégie de coopération de l’OMS avec les pays 2008–2013 Guinée Equatoriale 2009 http://www.who.int/countryfocus/cooperation_strategy/ccs_gnq_fr.pdf23634464

[B10] Biedron C Pagano M Hedt BL Kilian A Ratcliffe A Mabunda S Valadez JJ An assessment of Lot Quality Assurance Sampling to evaluate malaria outcome indicators: extending malaria indicator surveys Int J Epidemiol 2010 39 72 79 10.1093/ije/dyp363 20139435 PMC2912491

[B11] Cibulskis RE Bell D Christophel EM Hii J Delacollette C Bakyaita N Aregawi MW Estimating trends in the burden of malaria at country level AmJTrop Med Hyg 2007 77 133 137 18165485

[B12] Reddy MR Godoy A Dion K Matias A Callender K Kiszewski AE Kleinschmidt I Ridl FC Powell JR Caccone A Slotman MA Insecticide resistance allele frequencies in Anopheles gambiae before and after anti-vector interventions in continental Equatorial Guinea Am J Trop Med Hyg 2013 88 897 907 10.4269/ajtmh.12-0467 23438768 PMC3752755

[B13] Morassin B Fabre R Berry A Magnaval JF One year's experience with the polymerase chain reaction as a routine method for the diagnosis of imported malaria AmJTrop Med Hyg 2002 66 503 508 10.4269/ajtmh.2002.66.503 12201583

[B14] Bass C Nikou D Donnelly MJ Williamson MS Ranson H Ball A Vontas J Field LM Detection of knockdown resistance (kdr) mutations in *Anopheles gambiae:* a comparison of two new high-throughput assays with existing methods. Malar J 2007 6 111 10.1186/1475-2875-6-111 17697325 PMC1971715

[B15] Bass C Nikou D Vontas J Donnelly MJ Williamson MS Field LM The vector population monitoring tool (VPMT): High-throughput DNA-based diagnostics for the monitoring of mosquito vector populations Malar Res Treat 2010 2010 190434 22347668 10.4061/2010/190434 PMC3276000

[B16] United Nations Childrens Fund Malaria & children, progress in intervention coverage, summary update 2009 http://www.unicef.org/health/files/WMD_optimized_reprint.pdf23658824

[B17] StataCorp: Stata Release 12. Statistical Software 2011 College Station, TX: StataCorp LP

[B18] Stata Press Stata Survey Data Reference Manual, Release 12 2011 College Station, TX: StataCorp LP

[B19] Athrey G Hodges TK Reddy MR Overgaard HJ Matias A Ridl FC Kleinschmidt I Caccone A Slotman MA The effective population size of malaria mosquitoes: large impact of vector control PLoS Genet 2012 8 e1003097 10.1371/journal.pgen.1003097 23271973 PMC3521722

[B20] Lengeler C Insecticide-treated bed nets and curtains for preventing malaria Cochrane Database Syst Rev 2004 CD000363 15106149 10.1002/14651858.CD000363.pub2

[B21] Steketee RW Campbell CC Impact of national malaria control scale-up programmes in Africa: magnitude and attribution of effects Malar J 2010 9 299 10.1186/1475-2875-9-299 20979634 PMC2988827

[B22] Pluess B Tanser FC Lengeler C Sharp BL Indoor residual spraying for preventing malaria Cochrane Database Syst Rev 2010 CD006657 10.1002/14651858.CD006657 20393950 PMC6532743

[B23] Kleinschmidt I Schwabe C Shiva M Segura JL Sima V Mabunda SJ Coleman M Combining indoor residual spraying and insecticide-treated net interventions AmJTrop Med Hyg 2009 81 519 524 PMC3836236 19706925

[B24] Moreno M Vicente JL Cano J Berzosa PJ de Lucio A Nzambo S Bobuakasi L Buatiche JN Ondo M Micha F Do Rosario VE Pinto J Benito A Knockdown resistance mutations (kdr) and insecticide susceptibility to DDT and pyrethroids in *Anopheles gambiae* from Equatorial Guinea Trop Med Int Health 2008 13 430 433 10.1111/j.1365-3156.2008.02010.x 18397404

[B25] World Health Organization Global malaria control and elimination: report of a technical review 2008 Geneva

[B26] Bradley J Matias A Schwabe C Vargas D Monti F Nseng G Kleinschmidt I Increased risks of malaria due to limited residual life of insecticide and outdoor biting versus protection by combined use of nets and indoor residual spraying on Bioko Island. Equatorial Guinea Malar J 2012 11 242 10.1186/1475-2875-11-242 22835049 PMC3458978

