# Supplementary material for: Five years of malaria control in the continental region, Equatorial Guinea
Source: Malar J. 2013 May 7;12:154. doi: 10.1186/1475-2875-12-154 (PMC3652729; doi:10.1186/1475-2875-12-154)
Supplement: Additional file 1 — Use of LLIN the previous night in children aged 1-4 (percentage, [95% CI], N), Equatorial Guinea. Description: Data on the use of LLIN the previous night among children 1-4 years old broken down by sentinel site, province and by targeted intervention. [file 1475-2875-12-154-S1.pdf]

Use of LLIN the previous night in children aged 1-4 (percentage, [95% CI], N), Equatorial Guinea

	**Year**				
**Sentinel site**	**2007**	**2008**	**2009**	**2010**	**2011**
Akurenam	17.6% (10.7,27.4) 148	23.8% (13.7,38.1) 84	44.9% (32.8,57.7) 98	16.3% (9.1,27.6) 104	7.6% (4.0,13.9) 118
Bicurga	9.8% (4.5,20.0) 92	34.7% (22.8,48.9) 72	30.0% (19.8,42.6) 100	12.9% (6.5,24.1) 116	11.4% (5.6,21.9) 79
Niefang	12.3% (6.8,21.3) 122	31.5% (20.8,44.6) 89	35.7% (26.2,46.5) 112	21.0% (13.1,31.9) 100	9.2% (4.3,18.5) 87
**Centro Sur**	**13.8% (10.1,18.6) 362**	**29.8% (24.5,35.7) 245**	**36.8% (29.7,44.4) 310**	**16.6% (12.8,21.2) 320**	**9.2% (7.4,11.3) 284**
Ebebeyin	16.5% (9.6,27.0) 121	19.8% (11.2,32.4) 91	12.1% (7.0,20.0) 116	8.2% (3.8,16.8) 110	0.9% (0.1,6.1) 113
Mico Miseng	15.6% (9.1,25.4) 109	6.0% (1.9,17.3) 67	9.8% (4.9,18.6) 122	7.1% (2.6,17.8) 99	9.5% (4.0,21.0) 95
Nsok Nsomo	13.7% (6.8,25.6) 117	17.2% (8.6,31.5) 93	10.3% (4.6,21.5) 107	12.0% (6.4,21.2) 92	1.8% (0.5,7.1) 109
**Kie-Ntem**	**15.3% (13.8,16.9) 347**	**15.1% (9.6,23.1) 251**	**10.7% (9.5,12.0) 345**	**9.0% (6.8,11.8) 301**	**3.8% (1.1,12.2) 317**
Ayamiken	6.7% (0.9,35.9) 15	14.3% (7.1,26.5) 70	2.9% (0.7,10.7) 70	9.0% (4.2,18.1) 89	3.0% (0.4,18.3) 67
Etofili	32.1% (21.8,44.5) 137	23.1% (13.2,37.1) 117	18.1% (11.0,28.3) 116	20.2% (11.5,33.0) 104	18.3% (10.5,29.9) 104
Kogo	28.6% (18.5,41.3) 84	18.5% (9.8,32.1) 81	17.2% (10.4,27.1) 99	11.4% (5.9,20.7) 88	15.3% (8.5,26.0) 98
Mbini	31.3% (21.4,43.3) 99	23.7% (12.6,40.0) 76	10.6% (5.7,19.0) 94	17.6% (10.2,28.9) 85	12.5% (6.4,23.0) 80
Ngolo	11.9% (6.5,20.9) 151	13.5% (6.7,25.2) 104	8.6% (4.2,17.0) 116	15.2% (8.6,25.5) 112	14.3% (7.7,24.9) 119
Ukomba	27.7% (18.1,40.0) 119	15.5% (7.9,28.1) 110	21.3% (12.6,33.7) 94	22.4% (13.2,35.5) 107	11.7% (6.0,21.6) 111
Yengue	26.2% (12.3,47.3) 42	26.5% (12.8,47.0) 34	7.5% (2.3,21.6) 40	26.7% (12.2,48.7) 30	10.0% (3.2,27.0) 30
**Litoral**	**25.0% (18.0,33.8) 647**	**18.6% (15.2,22.5) 592**	**13.2% (9.0,18.9) 629**	**16.7% (13.0,21.2) 615**	**13.0% (10.0,16.7) 609**
Akonibe	23.6% (14.2,36.5) 123	30.1% (19.6,43.2) 93	59.3% (47.6,70.0) 118	36.6% (26.4,48.1) 112	19.6% (12.4,29.8) 112
Anisok	25.7% (17.2,36.5) 140	15.4% (9.3,24.3) 117	42.0% (32.1,52.6) 138	20.2% (11.9,32.2) 94	9.8% (4.7,19.5) 122
Mongomo	16.3% (9.4,26.8) 153	13.4% (7.2,23.5) 127	30.6% (20.8,42.6) 124	0.0% 0	12.6% (6.7,22.4) 119
Nsork	14.2% (8.0,23.8) 106	19.5% (10.6,33.2) 82	45.5% (36.1,55.2) 143	37.2% (26.1,49.9) 94	23.2% (14.1,35.8) 99
**Wele-Nzas**	**20.1% (15.4,25.8) 522**	**18.9% (12.9,26.7) 419**	**44.2% (34.2,54.6) 523**	**31.7% (22.9,41.9) 300**	**15.9% (11.0,22.5) 452**
**No intervention**	19.7% (16.1,23.9) 1878	17.5% (13.0,23.1) 670	4.5% (2.2,9.3) 110	13.4% (6.0,27.3) 119	0.0% 0
**LLINs distributed**	0.0% 0	29.8% (24.5,35.7) 245	41.4% (34.1,49.2) 833	23.9% (16.3,33.6) 620	13.3% (9.5,18.4) 736
**IRS received**	0.0% 0	18.6% (15.2,22.5) 592	13.3% (10.4,16.9) 864	14.3% (10.6,19.0) 797	9.8% (6.2,15.1) 926

Province total rows are shown in bold. MIS were carried out at 17 sentinel sites between April and June of each year from 2007 to 2011. LLINs were distributed in Centro Sur Province from June through August 2007 (after the MIS) and subsequently in Wele-Nzas Province from August through October 2008. A first round of indoor residual spraying was commenced in Litoral Province in June 2007 and was completed in December 2007. From March 2008 through September 2008, a second round of IRS was conducted in Litoral Province and a first round was conducted in Kie Ntem Province. From 2009 through 2011, due to a reorganization of the spray plan, two rounds of IRS were conducted in each province, the first occurring from February through March, and the second from August through September. The row titled “No intervention” therefore includes all sentinel sites in 2007 and sites in Kie-Ntem and Wele-Nzas in 2008. In 2009 and 2010 Ayamiken and Yengue did not receive IRS and were included in the row “No intervention”.
