# Supplementary material for: Five years of malaria control in the continental region, Equatorial Guinea
Source: Malar J. 2013 May 7;12:154. doi: 10.1186/1475-2875-12-154 (PMC3652729; doi:10.1186/1475-2875-12-154)
Supplement: Additional file 2 — Percentage of households reporting IRS in the last six months (percentage, [95% CI], N), Equatorial Guinea. Description: Data on household IRS coverage broken down by sentinel site, province and by targeted intervention. [file 1475-2875-12-154-S2.pdf]

Percentage of households reporting IRS in the last six months (percentage, [95% CI], N), Equatorial Guinea

Sentinel site	Year				
	**2007**	**2008**	**2009**	**2010**	**2011**
Akurenam	0.0% 95	2.4% (0.6,9.1) 83	1.1% (0.2,7.5) 89	0.0% 91	0.0% 101
Bicurga	1.8% (0.3,11.8) 55	2.3% (0.6,8.8) 86	0.0% 82	4.7% (1.8,11.9) 85	0.0% 73
Niefang	0.0% 73	0.0% 87	0.0% 92	0.0% 87	4.3% (1.6,10.8) 94
**Centro Sur**	**0.4% (0.1,3.3) 223**	**1.6% (0.6,3.8) 256**	**0.4% (0.1,2.2) 263**	**1.5% (0.2,8.9) 263**	**1.5% (0.3,8.2) 268**
Ebebeyin	1.3% (0.2,8.8) 76	1.1% (0.2,7.7) 87	58.6% (48.0,68.5) 87	54.5% (44.1,64.6) 88	67.7% (57.6,76.4) 93
Mico Miseng	0.0% 75	0.0% 84	59.8% (49.5,69.3) 92	59.0% (47.8,69.3) 78	72.5% (61.7,81.2) 80
Nsok Nsomo	0.0% 62	0.0% 86	81.6% (72.1,88.4) 87	61.0% (49.8,71.2) 77	73.6% (63.3,81.8) 87
**Kie-Ntem**	**0.5% (0.1,2.6) 213**	**0.4% (0.1,2.3) 257**	**66.5% (52.4,78.2) 266**	**58.0% (54.5,61.5) 243**	**71.2% (67.7,74.4) 260**
Ayamiken	0.0% 16	63.0% (52.0,72.7) 81	43.8% (30.5,57.9) 48	22.2% (14.5,32.5) 81	26.0% (17.4,36.9) 77
Etofili	6.0% (2.5,13.5) 84	27.6% (19.2,37.9) 87	39.3% (29.8,49.8) 89	48.8% (38.3,59.4) 84	42.7% (32.5,53.6) 82
Kogo	2.6% (0.6,9.7) 78	16.1% (9.8,25.4) 87	53.0% (42.3,63.5) 83	58.8% (47.7,69.0) 80	66.7% (55.8,76.0) 81
Mbini	0.0% 91	9.5% (4.8,17.9) 84	57.1% (46.4,67.3) 84	43.9% (33.6,54.8) 82	52.8% (42.5,62.9) 89
Ngolo	2.2% (0.6,8.5) 89	32.5% (23.0,43.7) 77	42.5% (32.6,53.1) 87	45.6% (35.0,56.6) 79	44.3% (34.3,54.8) 88
Ukomba	2.4% (0.6,9.1) 83	19.3% (12.4,28.9) 88	77.8% (42.1,94.4) 9	40.7% (31.1,51.0) 91	52.0% (42.3,61.6) 100
Yengue	0.0% 23	51.0% (37.3,64.6) 49	51.7% (34.1,68.9) 29	27.3% (12.8,48.9) 22	60.9% (40.2,78.2) 23
**Litoral**	**2.4% (1.1,5.0) 464**	**29.7% (17.4,45.8) 553**	**48.3% (41.6,54.9) 429**	**42.6% (33.6,52.0) 519**	**48.3% (38.7,58.1) 540**
Akonibe	0.0% 94	0.0% 87	1.1% (0.1,7.1) 95	0.0% 94	2.1% (0.5,8.1) 94
Anisok	1.1% (0.2,7.2) 93	2.4% (0.6,9.2) 82	3.6% (1.2,10.5) 84	0.0% 81	1.1% (0.2,7.5) 90
Mongomo	5.1% (1.9,12.9) 78	1.3% (0.2,8.9) 75	1.1% (0.2,7.5) 90	0.0% 96	4.1% (1.5,10.4) 98
Nsork	0.0% 91	0.0% 88	1.5% (0.4,5.6) 137	0.0% 84	3.3% (1.1,9.8) 90
**Wele-Nzas**	**1.4% (0.3,6.2) 356**	**0.9% (0.3,3.1) 332**	**1.7% (1.0,3.0) 406**	**0.0% 355**	**2.7% (1.7,4.3) 372**
**No intervention**	1.4% (0.7,2.9) 1256	0.7% (0.2,2.0) 589	46.8% (41.0,52.6) 77	23.3% (20.8,26.0) 103	0.0% 0
**LLINs distributed**	0.0% 0	1.6% (0.6,3.8) 256	1.2% (0.6,2.4) 669	0.6% (0.1,4.8) 618	2.2% (1.2,4.1) 640
**IRS received**	0.0% 0	29.7% (17.4,45.8) 553	56.3% (45.7,66.4) 618	51.3% (45.6,56.9) 659	55.8% (45.5,65.5) 800

Province total rows are shown in bold. MIS were carried out at 17 sentinel sites between April and June of each year from 2007 to 2011. LLINs were distributed in Centro Sur Province from June through August 2007 (after the MIS) and subsequently in Wele-Nzas Province from August through October 2008. A first round of indoor residual spraying was commenced in Litoral Province in June 2007 and was completed in December 2007. From March 2008 through September 2008, a second round of IRS was conducted in Litoral Province and a first round was conducted in Kie Ntem Province. From 2009 through 2011, due to a reorganization of the spray plan, two rounds of IRS were conducted in each province, the first occurring from February through March, and the second from August through September. The row titled “No intervention” therefore includes all sentinel sites in 2007 and sites in Kie-Ntem and Wele-Nzas in 2008. In 2009 and 2010 Ayamiken and Yengue did not receive IRS and were included in the row “No intervention”.
