# Supplementary material for: Five years of malaria control in the continental region, Equatorial Guinea
Source: Malar J. 2013 May 7;12:154. doi: 10.1186/1475-2875-12-154 (PMC3652729; doi:10.1186/1475-2875-12-154)
Supplement: Additional file 3 — Prevalence of P. falciparum in children aged 1-4 (percentage, [95% CI], N), Equatorial Guinea. Description: Data on prevalence of P. falciparum in children aged 1-4 years broken down by sentinel site, province and by targeted intervention. [file 1475-2875-12-154-S3.pdf]

Prevalence of *P. falciparum* in children aged 1-4 (percentage, [95% CI], N), Equatorial Guinea

**Sentinel site**	**Year**				
	**2007**	**2008**	**2009**	**2010**	**2011**
Akurenam	54.5% (45.8,62.9) 145	49.3% (36.6,62.1) 71	52.2% (40.5,63.6) 92	77.7% (67.8,85.2) 103	62.6% (53.0,71.3) 107
Bicurga	81.3% (70.6,88.7) 91	58.8% (44.8,71.6) 68	67.4% (56.7,76.5) 95	73.0% (63.5,80.7) 111	53.8% (42.7,64.7) 78
Niefang	69.6% (58.8,78.6) 115	72.6% (61.0,81.8) 84	69.6% (58.5,78.8) 102	70.5% (60.1,79.1) 105	57.3% (45.4,68.4) 89
**Centro Sur**	**66.4% (51.5,78.6) 351**	**61.0% (47.9,72.7) 223**	**63.3% (53.3,72.3) 289**	**73.7% (69.8,77.2) 319**	**58.4% (53.7,63.0) 274**
Ebebeyin	90.8% (83.9,95.0) 109	78.3% (66.3,86.9) 83	71.3% (61.2,79.6) 108	75.0% (66.1,82.2) 108	35.5% (26.6,45.5) 107
Mico Miseng	57.3% (45.8,68.1) 103	73.8% (61.6,83.1) 61	49.1% (39.9,58.4) 112	52.6% (42.8,62.2) 97	38.7% (29.1,49.3) 93
Nsok Nsomo	76.8% (66.9,84.4) 112	62.5% (50.5,73.1) 88	57.3% (47.3,66.7) 103	65.6% (54.0,75.6) 96	51.9% (42.9,60.7) 108
**Kie-Ntem**	**75.3% (55.0,88.4) 324**	**71.1% (61.1,79.4) 232**	**59.1% (47.1,70.2) 323**	**64.8% (52.3,75.5) 301**	**42.2% (33.3,51.6) 308**
Ayamiken	86.7% (58.4,96.8) 15	82.8% (72.1,90.0) 64	78.7% (65.4,87.8) 61	87.9% (79.8,93.0) 99	80.3% (67.3,89.0) 66
Etofili	41.4% (32.6,50.8) 128	30.4% (19.8,43.7) 69	43.6% (34.0,53.7) 117	39.8% (31.7,48.5) 108	32.3% (23.1,43.1) 96
Kogo	51.3% (38.3,64.2) 76	56.3% (44.6,67.2) 80	52.0% (41.3,62.5) 100	73.3% (63.1,81.4) 86	42.6% (31.5,54.4) 94
Mbini	71.0% (61.5,78.9) 93	72.6% (60.6,82.1) 73	72.6% (60.8,81.9) 84	84.9% (76.0,90.9) 86	61.5% (48.7,72.9) 78
Ngolo	70.6% (61.1,78.6) 136	35.4% (25.2,47.2) 79	65.0% (55.8,73.2) 120	69.6% (59.9,77.7) 115	53.0% (43.3,62.5) 117
Ukomba	37.8% (27.8,49.0) 111	33.3% (23.9,44.4) 84	50.0% (39.2,60.8) 90	54.8% (44.1,65.1) 104	22.1% (15.3,30.9) 104
Yengue	82.9% (68.7,91.5) 41	81.8% (63.1,92.2) 33	81.1% (60.7,92.3) 37	93.8% (79.3,98.3) 32	75.9% (46.7,91.8) 29
**Litoral**	**57.2% (42.8,70.4) 600**	**52.9% (36.4,68.8) 482**	**59.9% (49.2,69.8) 609**	**68.7% (52.5,81.4) 630**	**47.8% (33.3,62.7) 584**
Akonibe	54.2% (45.5,62.7) 118	65.6% (54.0,75.5) 90	41.7% (32.5,51.6) 115	70.0% (60.3,78.2) 110	44.4% (35.3,54.0) 108
Anisok	85.8% (78.2,91.1) 134	66.7% (54.5,76.9) 111	54.5% (44.4,64.3) 121	80.8% (71.6,87.5) 99	83.9% (74.6,90.2) 118
Mongomo	85.3% (77.9,90.5) 143	79.5% (69.9,86.6) 117	63.2% (53.9,71.7) 117	66.1% (55.7,75.1) 112	51.8% (41.4,62.0) 112
Nsork	75.0% (66.6,81.8) 100	75.9% (65.5,84.0) 79	55.2% (46.0,64.1) 143	76.3% (65.5,84.6) 93	64.3% (53.3,73.9) 98
**Wele-Nzas**	**76.0% (59.4,87.2) 495**	**72.0% (64.7,78.4) 397**	**53.8% (45.9,61.5) 496**	**72.9% (66.3,78.7) 414**	**61.5% (43.7,76.7) 436**
**No intervention**	67.6% (58.0,75.9) 1770	71.7% (65.9,76.9) 629	79.6% (77.8,81.3) 98	89.3% (85.5,92.2) 131	Not applicable
**LLINs distributed**	Not applicable	61.0% (47.9,72.7) 223	57.3% (50.1,64.3) 785	73.3% (69.2,77.0) 733	60.3% (49.2,70.4) 710
**IRS received**	Not applicable	52.9% (36.4,68.8) 482	57.3% (49.4,64.9) 834	63.9% (52.9,73.6) 800	45.9% (35.8,56.3) 892

Province total rows are shown in bold. MIS were carried out at 17 sentinel sites between April and June of each year from 2007 to 2011. LLINs were distributed in Centro Sur Province from June through August 2007 (after the MIS) and subsequently in Wele-Nzas Province from August through October 2008. A first round of indoor residual spraying was commenced in Litoral Province in June 2007 and was completed in December 2007. From March 2008 through September 2008, a second round of IRS was conducted in Litoral Province and a first round was conducted in Kie Ntem Province. From 2009 through 2011, due to a reorganization of the spray plan, two rounds of IRS were conducted in each province, the first occurring from February through March, and the second from August through September. The row titled “No intervention” therefore includes all sentinel sites in 2007 and sites in Kie-Ntem and Wele-Nzas in 2008. In 2009 and 2010 Ayamiken and Yengue did not receive IRS and were included in the row “No intervention”.
